# Deducing cardiorespiratory motion of cardiac substructures using a novel 5D-MRI workflow for radiotherapy

**DOI:** 10.1088/1361-6560/ae5752

**Published:** 2026-04-29

**Authors:** Chase Ruff, Tarun Naren, Oliver Wieben, Prashant Nagpal, Kevin Johnson, Jiwei Zhao, Thomas Grist, Andrew Baschnagel, Carri Glide-Hurst

**Affiliations:** 1 Department of Medical Physics, University of Wisconsin-Madison, Madison, WI, United States of America; 2 Department of Human Oncology, University of Wisconsin-Madison, Madison, WI, United States of America; 3 Department of Radiology, University of Wisconsin-Madison, Madison, WI, United States of America; 4 Department of Biostatistics and Medical Informatics, University of Wisconsin-Madison, Madison, WI, United States of America; 5 Department of Statistics, University of Wisconsin-Madison, Madison, WI, United States of America

**Keywords:** magnetic resonance imaging, motion management, radiation therapy, cardiac, respiratory motion

## Abstract

*Objective.* Cardiotoxicity is a devastating complication of thoracic radiotherapy. However, current practice ignores the radiosensitivities and complex motion trajectories of individual substructures. Current imaging protocols in radiotherapy are insufficient to decouple and quantify cardiac motion, limiting substructure-specific motion considerations in treatment planning. We propose a 5D-MRI workflow for comprehensive substructure-specific motion analysis. *Approach.* Our 5D-MRI workflow was implemented in 10 healthy subjects (23–65 years) and two patients with lung cancer (67–69 years), with iterative reconstruction at end-exhale/inhale and active-exhale/inhale for end-systole/diastole. For motion assessment, proximal coronary arteries, chambers, great vessels, and cardiac valves/nodes were contoured across all images and verified. Centroid/bounding box excursion was calculated for cardiac, respiratory, and hysteresis motion. Distance metrics were tested for statistical independence across substructure pairings. Three thoracic radiotherapy plans were retrospectively analyzed using volunteer-derived internal organ-at-risk volumes (IRVs). Cardiac substructure motion was compared between volunteer and patient cohorts. *Main results.* 5D-MRI images were successfully acquired and contoured for all volunteers. Cardiac motion exceeded 1 cm for right-heart substructures and was greatest for the right coronary artery. Respiratory motion was largest for the inferior vena cava/left ventricle. Respiratory hysteresis was generally <5 mm but >5 mm for some subjects. For cardiac motion, statistically significant differences were observed between coronary arteries/chambers/great vessels and between right/left-sided substructures. Respiratory motion differed significantly between the heart base/apex. For three plans, D_0.03cc_ increased by up to 21.5 Gy across volunteer-derived cardiorespiratory IRVs. Patients’ right-heart motion ranged from 7–19 mm, yet left-heart motion varied due to tumor location. *Significance.* Our 5D-MRI workflow successfully decouples cardiorespiratory motion in a ∼5 min free-breathing acquisition. Cardiac motion was >5 mm for coronary arteries/chambers, while respiratory motion was >5 mm for all substructures. Statistically significant differences were observed between cardiac substructures for cardiac and respiratory motion. The interplay between tumor location and motion magnitude affected substructure dose.

## 1. Introduction

A devastating complication of radiotherapy for lung, breast, esophageal, and other thoracic cancers is cardiotoxicity, which can result in congestive heart failure, coronary artery disease, and pericardial effusion (Ng 2011, Hardy *et al* 2010, Banfill *et al* 2021). The risk of having a major coronary event increases by 7.4% per additional Gy of mean heart dose during radiation treatments (Darby *et al* 2013), and increased dose to the left coronary artery (LCA) results in a higher risk of coronary artery disease following treatment (Atkins *et al* 2021, Cai *et al* 2023). Managing cardiotoxicity in the treatment of lung cancer remains a challenge, with 20% of patients experiencing cardiac complications following treatment (Walls *et al* 2025). A contributing factor to cardiotoxicity is intrafraction motion, including cardiorespiratory motion, which is difficult to decouple and manage with traditional imaging techniques (Bertholet *et al* 2019). Higher doses to specific substructures, namely the coronary arteries (Atkins *et al* 2021, 2024, Cai *et al* 2023), cardiac valves (Lee and Hahn 2023) and nodes (Kim *et al* 2022), left ventricle (Jacob *et al* 2019), and pulmonary vessels (Atkins *et al* 2024) have been correlated with specific cardiotoxicities. Thus, a recent thrust has been made to develop techniques for automated cardiac substructure segmentation and to estimate doses to each unique substructure for potential risk assessment and outcome modeling (Morris *et al* 2020a, Morris *et al* 2020b, Eber *et al* 2023, Finnegan *et al* 2023, Van Der Pol *et al* 2023, Summerfield *et al* 2024).

Generally, cardiac motion is not considered when calculating planning organ-at-risk margins (PRVs) for the heart in external beam radiotherapy (Morris *et al* 2021). However, respiratory motion of the heart is typically managed using gating techniques such as deep inspiration breath holds (DIBH), surface-guided imaging, or tracking respiratory motion of fiducials (Jagsi *et al* 2007, Alderliesten *et al* 2013). Current guidance recommends employing a 5 mm threshold for motion management, where motion management techniques should be considered if motion exceeds this threshold in any one direction (Keall *et al* 2006). Research has shown that cardiac motion differs across cardiac substructures and varies locally, with regions near the base of the heart and specific substructures experiencing displacements greater than 5 mm (Ouyang *et al* 2020) and even up to 1–2 cm (Yang *et al* 2023). Despite exceeding the motion management threshold, cardiac motion management is often unachievable in radiation oncology, due to the current lack of cardiac gating capabilities in imaging, treatment planning, and during radiation delivery. Numerous studies have shown that a single PRV margin fails to capture the full range of motion of cardiac substructures, indicating a need for robust, accurate motion quantification for thoracic radiotherapy (Li *et al* 2018, Tong *et al* 2018, Yang *et al* 2023).

An emerging cardiac application in radiotherapy that has shown significant promise is the treatment of ventricular tachycardia (VT) with stereotactic body radiation therapy (SBRT) (Robinson *et al* 2019, Petzl *et al* 2024). However, cardiotoxicities have been reported following SBRT, as clinical target volume (CTV) margins are typically expanded isotropically to account for motion, exposing unnecessary tissue to radiation (Robinson *et al* 2019, Petzl *et al* 2024). Thus, measurement and management of cardiorespiratory motion remain a challenge for VT SBRT procedures. Whether the heart is being spared in thoracic cancer radiotherapy or treated using SBRT for VT, quantifying cardiorespiratory motion and limiting dose to healthy cardiac tissue to reduce cardiotoxicity remain an unmet need.

Cardiac MRI is routinely used in diagnostic imaging to evaluate cardiac anatomy, function, and blood flow (Rajiah *et al* 2023), and provides sufficient temporal resolution to accurately quantify cardiac motion (Tong *et al* 2018, Yuan *et al* 2019). The use of cardiac MRI in radioablation of atrial fibrillation and in treatment planning has been explored (Lydiard *et al* 2021, Bertelsen *et al* 2022), and as MRI simulation becomes increasingly available to clinics, incorporating cardiac MRI into routine radiation oncology workflows becomes possible. Building on our previous preliminary work, we implement a novel 5D-MRI workflow (Naren *et al* 2023, Ruff *et al* 2023, Ruff *et al* 2025), including reconstructions for both cardiac and respiratory phases from a single MRI acquisition, to decouple and quantify cardiorespiratory motion. Further, we apply 5D-MRI-derived internal organ-at-risk volumes (IRVs) to three thoracic radiotherapy cases to simulate how cardiac and respiratory motion affect endpoints of cardiac substructures. A prospective analysis of 5D-MRI is demonstrated for two patients with non-small cell lung cancer (NSCLC). The results of this work may be used for cardiac-spared treatment planning in NSCLC with potential extension to cardioprotection in targeted VT SBRT.

## 2. Methods and materials

### 2.1. Image acquisition and reconstruction

**Figure 1.**
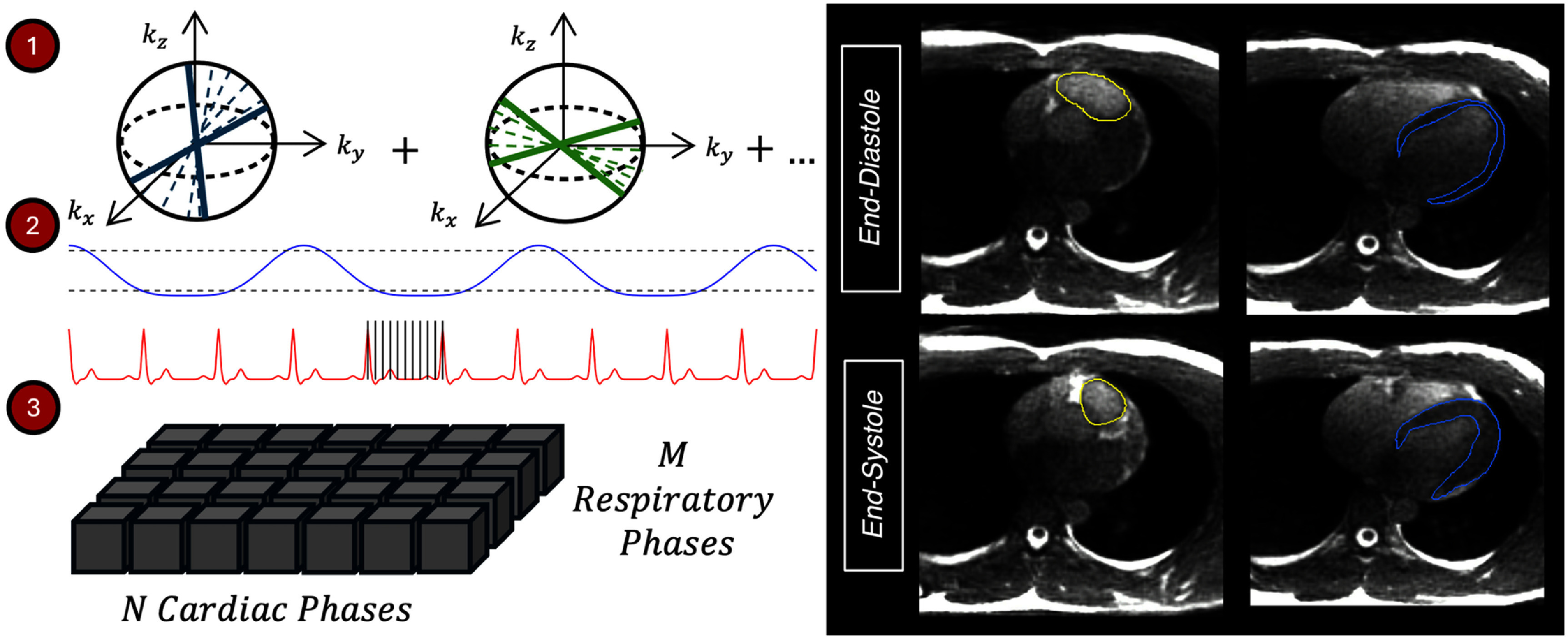
(Left) schematic for our 5D-MRI workflow where a 3D, radially sampled, bSSFP cardiac MRI is used for acquisition (1), with respiratory bellows and pulse oximeter signals used for retrospective reconstruction (2). Amplitude-based respiratory binning with M phases (dashed lines), and phase-based cardiac binning with N phases (solid lines), yields N x M reconstructed image volumes per dataset (3). (Right) example 5D-MRI images for end-exhale, end-systole/diastole, where changes in RV shape and position (yellow) and LV blood pool/myocardium (blue) are observed.

An overview of our novel 5D-MRI workflow, where data is binned across cardiac and respiratory phases from one, continuous native acquisition, is shown in figure 1. Data were acquired with a 3D radial balanced steady-state free precession (bSSFP) cardiac MRI sequence with the following parameters: 80 000 radial readouts, echo time/repetition time of 0.9/3.6 ms, flip angle of 35°, imaging volume of (40.0 cm)^3^, acquired and reconstructed isotropic resolution of 1.56 × 1.56 × 1.56 mm^3^, and scan time of ∼5 min on a 1.5 T clinical MRI-simulator (SIGNA Artist, GE Healthcare, Waukesha, WI). Respiratory bellows and pulse oximeter signals acquired during the scan were used for offline retrospective respiratory and cardiac sorting. Data were binned and reconstructed for 10 cardiac phases and 4 respiratory phases (end-inhale (top 10% of the respiratory waveform), end-exhale (bottom 10%), active inhale, and active exhale), where active inhale and active exhale were defined by the remaining 80% of the respiratory waveform between end-exhale and end-inhale. Advanced iterative local low rank reconstruction (Trzasko *et al* 2011) was used to mitigate undersampling artifacts. The resulting 5D dataset consisted of 40 image volumes for each subject (10 cardiac phases across 4 respiratory phases). A supplemental 3D bSSFP fat-saturated cardiac-gated sequence with navigator-based respiratory gating (3D-Nav) acquired at end-exhale was also included for each volunteer to bolster segmentation ability of the cardiac substructures with the following parameters: echo time/repetition time of 1.8/3.9 ms, flip angle of 70°, imaging volume of (35.0 cm)^3^–(38.0 cm)^3^, acquired/reconstruction resolution of 1.70 × 1.70 × 1.80 mm^3^/0.74 × 0.74 × 0.90 mm^3^, and scan time of 5–9 min. 10 healthy volunteers (9 M/1 F, 23–65 years old) were recruited for this study and scanned after providing informed consent.

### 2.2. Motion analysis

#### 2.2.1. Segmentation

A 15 substructure cardiac model was adopted for motion analysis, including the chambers (left/right ventricle (LV/RV), left/right atrium (LA/RA)), coronary arteries (right (RCA), left main (LMCA), left circumflex (LCX), and left anterior descending (LADA) arteries), ascending/descending aorta (AA/DA), pulmonary veins/artery (PVs/PA), superior/inferior vena cava (SVC/IVC), and whole heart (WH). All substructures were first manually delineated on the end-exhale, end-diastole phase of 5D-MRI following well-established radiotherapy contouring atlas definitions (Feng *et al* 2011, Duane *et al* 2017) and referring to the 3D-Nav as a reference to augment segmentation accuracy when needed. The proximal segments of the RCA, LADA, and LCX were delineated as regions near the base of the heart are most mobile during the cardiac cycle (Shechter *et al* 2006) and have been reported to be the most radiosensitive (Belzile‐Dugas and Eisenberg 2021). Contours were then propagated to the remaining 5D-MRI phases using a custom deformable image registration workflow in MIM Maestro (MIM Software, Cleveland, OH), and all propagated contours were visually inspected and modified to match the underlying anatomy. For the AA/DA, IVC/SVC, and PA/PVs, contour volume was conserved by using consistent superior/inferior extents of each substructure across 5D-MRI phases to reduce potential delineation errors. This was not necessary for the proximal coronary arteries, chambers, and WH as the entire substructure was contoured. For consistent delineation of the proximal coronary artery branches across subjects, definitions from Duane *et al* (2017) and the American Heart Association (Austen *et al* 1975) were followed, with all arteries delineated using a 3 mm diameter brush. For each respiratory phase, contours were delineated for end-systole and end-diastole instead of all 10 cardiac phases as these two phases have been shown to be sufficient for calculating the full extent of cardiac motion (Kataria *et al* 2016, Li *et al* 2018). Final contours were verified by a cardiovascular radiologist with 10+ years of experience.

Emerging literature has suggested the importance of cardiac valves and nodes in cardiotoxicity, thus as an exploratory endpoint, a geometric model was adopted for the cardiac valves (mitral valve (V-MV), tricuspid valve (V-TV), aortic valve (V-AV), and pulmonary valve (V-PV)) and conduction nodes (sinoatrial node (SAN) and atrioventricular node (AVN)). Each valve was defined by an 8 mm isotropic expansion of the respective ventricle, followed by a masking (i.e. Boolean intersection) by the corresponding atrium/great vessel following the methods by Finnegan *et al* (2023) the V-MV and V-AV were defined by an expansion of the left ventricle and masking with the LA and AA, respectively. The V-TV and V-PV were defined by an expansion of the RV and masking with the RA and PA, respectively. The SAN and AVN were defined by a sphere with a 1 cm radius, placed at the intersection of the SVC/RA and cardiac chambers, respectively, as defined by Loap *et al* (Loap *et al* 2021, Finnegan *et al* 2023). While these are not direct segmentations, their inclusion in our analysis may provide insights on the mobility of these critical regions of interest.

#### 2.2.2. Centroid and bounding box analysis

The centroid of each contour was found in each image volume and used to calculate centroid displacements across both the respiratory and cardiac cycles for each volunteer, for the anterior–posterior (AP), right–left (RL), and superior–inferior (SI) directions, along with vector centroid displacements ($\sqrt {{\mathrm{A}}{{\mathrm{P}}^2} + {\mathrm{R}}{{\mathrm{L}}^2} + {\mathrm{S}}{{\mathrm{I}}^2}} $). Using in-house MATLAB functions (MATLAB 2021b, The MathWorks Inc., Natick, Massachusetts), 3D bounding boxes were automatically calculated for each of the cardiac substructures for each volunteer, throughout all cardiac and respiratory phases, to capture local displacements of each contour. The displacement of the right, left, posterior, anterior, inferior, and superior edges of the bounding boxes were calculated between phases. For both centroid and bounding box displacements, voxel displacements were converted to physical displacements by multiplying by the image resolution. Cardiac motion was defined as motion from end-diastole to end-systole averaged across respiratory states, while respiratory motion was defined as motion from end-exhale to end-inhale averaged across cardiac states. The maximum hysteresis of cardiac substructures due to respiration was calculated following Boldea *et al* where hysteresis was defined as the distance between pairs of points between exhalation and inhalation trajectories for lung tumors on 4-dimensional computed tomography (4D-CT) (2008). In our implementation, hysteresis was measured by finding the difference in both centroid and bounding box locations between active exhale and active inhale, where non-zero displacement between active exhale and inhale indicates hysteresis. Displacements due to respiratory hysteresis were averaged across all cardiac phases.

#### 2.2.3. Voxelwise respiratory and cardiac displacements

To describe voxelwise displacements, the distance to agreement between a reference contour ‘A’ and target contour ‘B’ was calculated pointwise (equation 1). Cardiac motion was calculated for each substructure with the reference contour ‘A’ defined at end-exhale, end-diastole and target contour ‘B’ defined at end-exhale, end-systole. Similarly, respiratory motion was calculated with reference contour ‘A’ defined at end-exhale, end-diastole, while target contour ‘B’ was defined at end-inhale, end-diastole,

\begin{equation*}{d_a} = \mathop {\min }\limits_{b \in B} \left\| {a - b} \right\|.\end{equation*}

From these displacements, the 95% Hausdorff distance (HD95) and mean distance to agreement (MDA) were calculated, defined by the 95th percentile and mean of the displacements, respectively.

#### 2.2.4. Statistical considerations

Statistical differences in cardiac and respiratory motion were tested for the physiological groupings of substructures (coronary arteries, cardiac chambers, great vessels, and WH), individual substructure pairs within each physiological group (e.g., RCA vs LADA for coronary arteries), substructures in the right heart (RCA, RA, RV) vs left heart (LADA, LMCA, LCX, LV, LA), substructures near the base (RCA, LADA, LMCA, LCX, AA, DA, PA, PVs, SVC) vs apex of the heart (LV, RV, LA, RA, IVC), RCA vs right chambers (RA/RV), and LCA vs left chambers (LV/LA), for 8 different distance metrics (centroid displacement in the AP, RL, and SI directions, centroid vector displacements, mean and maximum bounding box displacements, HD95, and MDA). All testing was performed in R using the Kruskal–Wallis nonparametric test with Bonferroni correction, followed by Dunn’s test, with a significance level of 0.05 (RStudio v2023.6.1.524, Posit Software PBC, Boston, MA).

### 2.3. Retrospective IRV derivation and dosimetric evaluation

To demonstrate clinical application of 5D-MRI, three thoracic radiotherapy cases were retrospectively reviewed. Two NSCLC cases and an MR-guided case of a secondary malignant neoplasm overlapping with the heart, treated on a ViewRay 0.35 T MR-Linac, were included for analysis. The two NSCLC cases included adenocarcinomas to the right-middle lung and left-lower lung and were treated with 60 Gy in 30 fractions, while the adaptive MR-Linac case was treated with 35 Gy in 5 fractions. The coronary arteries and chambers were selected for dosimetric analysis due to cardiac motion exceeding 5 mm. CT simulation scans for 3 thoracic cancer patients had coronary arteries and chambers manually contoured and used as the reference for dosimetric comparison (e.g. no margin/motion considered). ICRU Report 62 defines the internal target volume (ITV) as the CTV with an added internal margin (IM) to encompass all possible movements and variations in the target (International Commission on Radiation Units and Measurements 1999). Similarly, the IRV is defined as an organ-at-risk with an added IM, reflecting all possible variations in shape or size of the underlying structure. Similar work by Li *et al* (2018) and Kataria *et al* (2016) used maximum displacements from end-diastole to end-systole as the IM to derive IRVs for the coronary arteries. Following similar methods, to estimate the dosimetric impact of cardiac, respiratory, and combined cardiorespiratory motion for each substructure, maximum bounding box displacements were used to derive volunteer‐specific IRVs observed in each of the 10 healthy volunteers. For each volunteer, the maximal displacements in the superior, inferior, anterior, posterior, right, and left directions were used to define the IRV expansion. Cardiac motion was defined from end-diastole to end-systole, and respiratory motion was defined from end-inhale to end-exhale, capturing the full range of motion for each substructure. IRVs for cardiorespiratory motion were defined as the sum of cardiac and respiratory IRVs. Dose–volume histograms (DVHs) were generated and changes in D_0.03cc_ were calculated for each cardiac substructure by comparing reference contours with and without the addition of the volunteer‐specific IRVs for cardiac, respiratory, and cardiorespiratory motion. This approach represents a worst‐case motion envelope for each substructure.

### 2.4. Prospective 5D-MRI in patients with NSCLC

Two patients (69, female and 67, female) with locally advanced NSCLC were enrolled in the IRB-approved trial for cardiac substructures sparing with thoracic cancers (NCT07132918) after providing informed consent. The same imaging parameters were used for patient scans from the prior volunteer scans. In both scans, image acquisition occurred after the administration of intravenous gadoterate meglumine contrast as part of standard of care imaging and ECG-based cardiac gating was used. Acquiring 5D-MRI post-contrast offers a potential increase in signal from the cardiac blood pool and improvement of cardiac substructure visualization. 5D-MRI images acquired at end-inhale across the cardiac cycle were contoured with the coronary arteries, chambers, and great vessels. Images reconstructed at end-inhale were used for analysis to match the DIBH treatment schema. Centroid and bounding box displacement from end-diastole to end-systole were compared to the healthy volunteer cohort.

## 3. Results

### 3.1. Image reconstruction and segmentation

**Figure 2.**
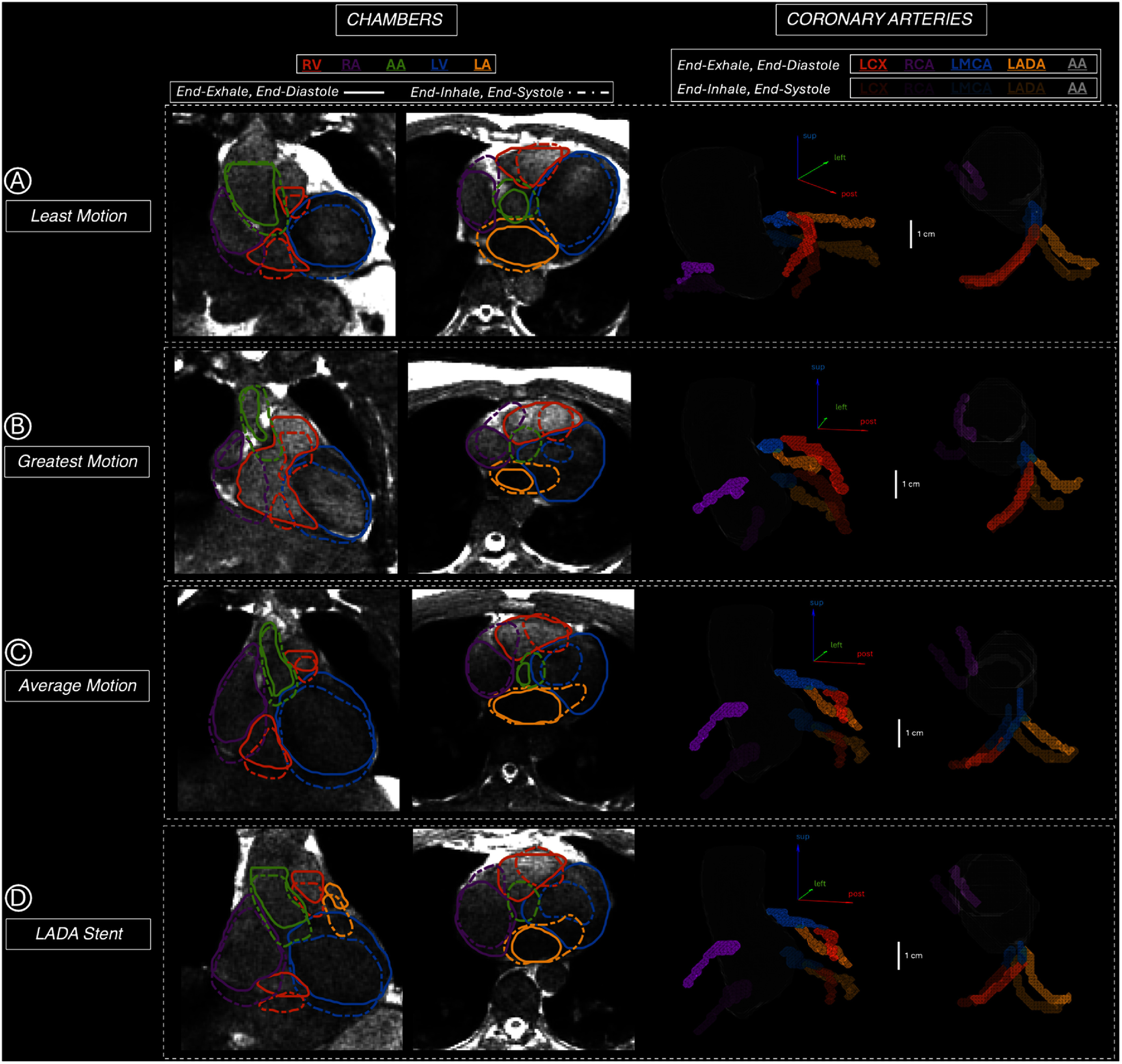
Contours for end-exhale, end-diastole (solid/bright) and end-inhale, end-systole (dashed/dark) on 5D-MRI are shown. Volunteers with the smallest (A), greatest (B), and average (C) magnitudes of cardiac motion, and a volunteer with an LADA coronary stent (D), are highlighted. Substructures generally move inferiorly, anteriorly, and left at end-inhale, end-systole compared to end-exhale, end-diastole. Locally, substructure displacements exceed 1 cm.

5D-MRI and 3D-Nav images were acquired for all 10 volunteers, and all 15 substructures were successfully contoured on end-diastole/end-systole across all 4 respiratory phases. Figure 2 highlights example 5D-MRI images and delineated chambers/coronary arteries for 4 select cases: subjects with the least, greatest, and average amount of cardiac motion, and a volunteer who had an LADA stent.

### 3.2. Excursion metrics

**Figure 3.**
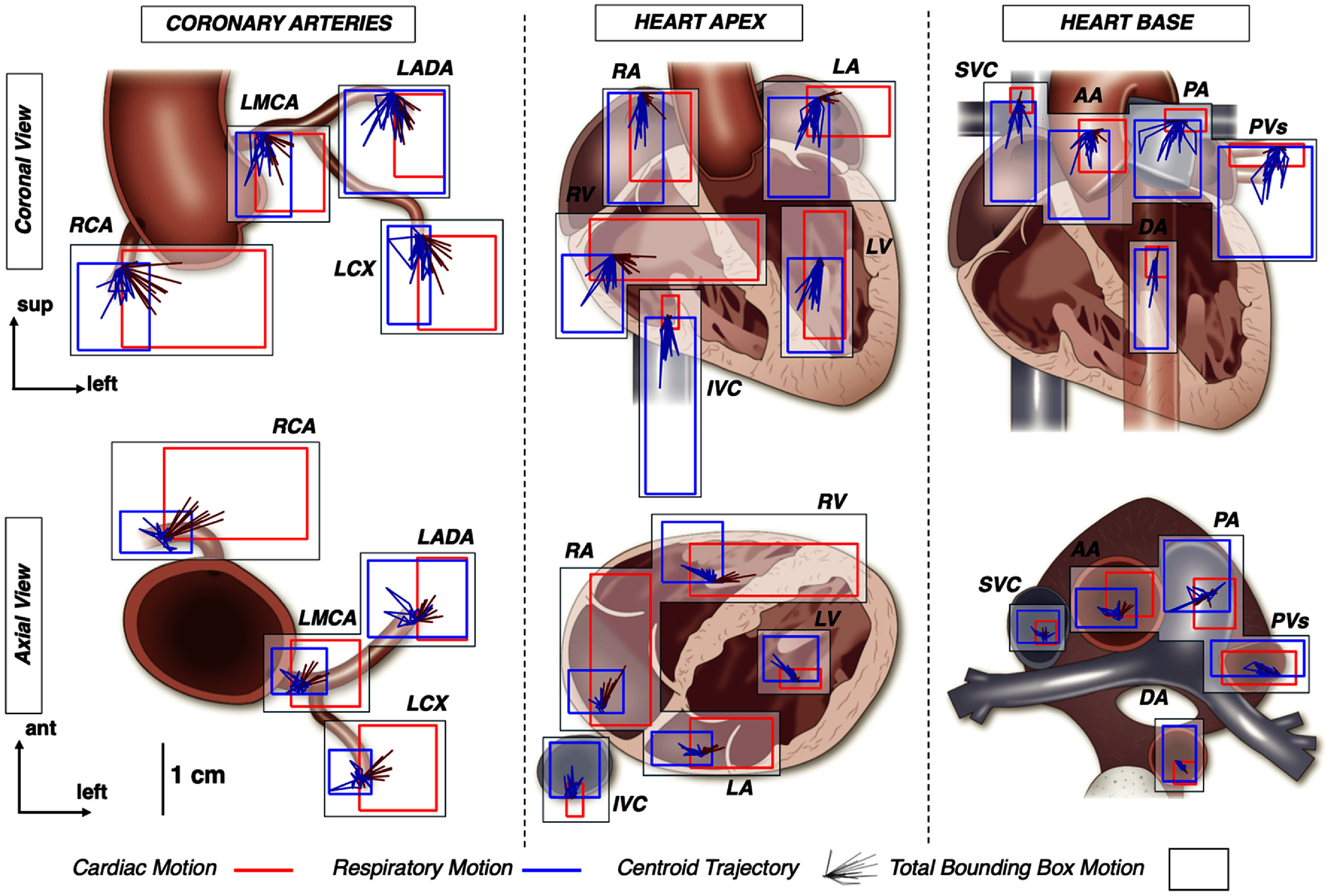
Cardiac (red) and respiratory (blue) trajectories for coronary arteries (left), heart apex (middle), and heart base (right), for coronal (top) and axial (bottom) views. Centroid trajectories are shown with a solid line for each volunteer, while total bounding box motion for the cohort is shown with a box.

Table 1 summarizes the mean AP, RL, SI, and vector centroid displacements for the cohort. Figure 3 highlights the centroid and bounding box excursions due to cardiac and respiratory motion of the volunteer cohort for each substructure. Centroid and bounding box motion of the cardiac valves and nodes are displayed in Table A1/A2. HD95 and MDA for all substructures are shown (Table A3). The number of volunteers with cardiac, respiratory, and hysteresis motion exceeding 5 mm for centroid/bounding box motion is shown (table 2).

**Table 1.** Centroid displacements across 10 subjects for cardiac and respiratory motion in RL, AP, SI directions, and vector displacements. Vector centroid displacement is greatest for the RCA for cardiac motion and the LV for respiratory motion. Motion >5 mm is bolded and italicized.

Cardiac and respiratory centroid motion for volunteer cohort									
		Cardiac motion				Respiratory motion			
		R–L (mm)	A–P (mm)	S–I (mm)	Vector (mm)	R–L (mm)	A–P (mm)	S–I (mm)	Vector (mm)
Coronary Arteries	RCA	***−5.5 ± 1.7***	3.8 ± 0.8	−3.6 ± 2.2	***8.2 ± 1.2***	0.5 ± 1.7	1.1 ± 1.1	−4.0 ± 1.5	4.6 ± 1.6
	LMCA	−2.3 ± 0.9	1.9 ± 1.0	−3.2 ± 2.2	4.8 ± 1.9	0.5 ± 1.3	0.8 ± 1.1	−4.5 ± 1.4	4.9 ± 1.5
	LADA	−2.0 ± 0.6	1.8 ± 1.3	−4.0 ± 1.8	***5.3 ± 1.4***	1.0 ± 2.1	1.4 ± 1.3	−4.9 ± 1.4	***5.8 ± 1.8***
	LCX	−3.0 ± 1.3	1.3 ± 1.5	−3.8 ± 1.9	***5.5 ± 1.8***	0.6 ± 0.9	0.3 ± 1.1	−4.6 ± 1.3	***5.0 ± 1.2***

Chambers	LV	0.0 ± 0.3	0.2 ± 0.3	−0.7 ± 0.4	0.9 ± 0.4	1.3 ± 1.1	1.6 ± 1.0	***−5.7 ± 1.2***	***6.2 ± 1.4***
	RV	−3.4 ± 1.0	0.5 ± 0.4	−1.2 ± 1.0	3.8 ± 1.0	1.1 ± 1.5	1.6 ± 0.5	−4.3 ± 1.2	***5.0 ± 1.4***
	LA	−1.6 ± 1.0	0.8 ± 0.3	−0.1 ± 0.5	1.9 ± 0.9	1.1 ± 1.1	0.5 ± 0.6	***−5.1 ± 1.3***	***5.3 ± 1.4***
	RA	−1.4 ± 0.9	3.4 ± 1.2	−1.4 ± 1.0	4.1 ± 1.4	0.1 ± 0.7	1.2 ± 0.9	***−5.2 ± 1.4***	***5.5 ± 1.3***

Great Vessels	AA	−0.6 ± 0.6	1.3 ± 0.6	−0.7 ± 0.7	1.8 ± 0.8	0.7 ± 1.2	0.7 ± 0.7	−3.6 ± 1.4	3.9 ± 1.5
	DA	0.0 ± 0.1	0.0 ± 0.3	0.3 ± 0.6	0.5 ± 0.5	0.6 ± 0.5	0.9 ± 0.3	−3.8 ± 1.1	3.9 ± 1.2
	PA	−0.2 ± 1.0	−0.2 ± 0.8	−0.3 ± 0.2	1.3 ± 0.5	1.1 ± 2.0	−0.1 ± 1.2	−4.0 ± 1.1	4.7 ± 1.6
	PVs	0.2 ± 0.8	0.2 ± 0.4	−0.3 ± 0.2	0.7 ± 0.7	1.3 ± 1.0	0.8 ± 0.6	***−5.3 ± 1.4***	***5.6 ± 1.5***
	IVC	−0.1 ± 0.2	0.1 ± 0.2	0.0 ± 0.2	0.4 ± 0.1	0.0 ± 0.7	2.3 ± 1.1	***−5.3 ± 1.8***	***5.9 ± 1.8***
	SVC	−0.3 ± 0.3	0.5 ± 0.4	0.0 ± 0.6	0.9 ± 0.5	0.0 ± 0.9	0.6 ± 0.7	−4.0 ± 1.3	4.2 ± 1.3

Whole Heart	WH	0.2 ± 0.2	−0.2 ± 0.2	0.4 ± 0.3	0.6 ± 0.3	1.2 ± 0.9	1.3 ± 0.6	−4.6 ± 0.9	***5.0 ± 1.0***

**Table 2.** Number of volunteers out of 10 with centroid (top) and bounding box (bottom) displacements >5 mm for cardiac, respiratory and hysteresis motion. Different volunteers exhibiting motion >5 mm for respiratory hysteresis are differentiated with asterisks.

Number of volunteers (out of 10) with cardiac and respiratory motion >5 mm																
		Coronary Arteries				Chambers				Great Vessels						WH
		RCA	LMCA	LADA	LCX	LV	RV	LA	RA	AA	DA	PA	PVs	IVC	SVC	WH
Centroid	Cardiac motion	10	3	5	6	0	1	0	1	0	0	0	0	0	0	0
	Respiratory motion	4	5	7	5	8	5	5	5	3	2	4	6	8	2	6
	Respiratory hysteresis	1*	0	0	1**	0	0	0	0	0	0	0	0	1***	0	0

Bounding Box	Cardiac motion	9	4	8	7	9	7	5	9	2	0	0	1	0	0	1
	Respiratory motion	8	8	10	10	9	9	10	10	8	10	9	9	10	8	8
	Respiratory hysteresis	1*	0	1**	1**	0	0	1***	0	0	0	0	1***	1***	0	0

#### 3.2.1. Cardiac motion

Isolated cardiac motion was highly substructure dependent. Across all substructures, cardiac motion was generally in the left, anterior, and inferior directions. Overall, the RCA was the most mobile substructure, with vector centroid motion >5 mm in all subjects and bounding box displacement >1 cm in the left direction. The LCA was additionally mobile, with 3/10–6/10 subjects having vector centroid displacements >5 mm and 4/10–8/10 with maximal bounding box displacements >5 mm (table 2). On average, MDA was >5 mm for the RCA, LADA, and LCX, with HD95 >9 mm for all coronary arteries. Centroid vector displacements were generally <5 mm for all chambers, while maximal bounding box displacements were >5 mm in 5/10–9/10 subjects (table 2). While MDA was <5 mm for all chambers, HD95 exceeded 5 mm for the RA and RV. The great vessels were the least mobile substructures, with vector centroid displacements <5 mm for all subjects and <1–2 mm on average. MDA and HD95 was <5 mm for all great vessels.

#### 3.2.2. Respiratory motion

Isolated respiratory motion varied less across substructures and was dominant in the SI direction, where centroid displacements were generally <1–2 mm in the AP and RL direction and >4–5 mm in the S–I direction (table 1). The most mobile substructures due to respiratory motion were the LV and IVC, with 8/10 subjects with a vector centroid displacement >5 mm for both substructures, while the least mobile were the SVC and DA (table 2). When considering bounding box motion, displacements were generally >5 mm for all substructures, with displacements >5 mm for at least 8/10 subjects. For the LCA, MDA was >5 mm, while HD95 was >5 mm for all substructures except the DA and WH for respiratory motion.

Motion due to respiratory hysteresis was generally <5 mm, aside from 3 separate volunteers who exhibited hysteresis motion >5 mm for the RCA, LADA/LCX, and LA/PVs/IVC, respectively.

#### 3.2.3. Cardiac valves and conduction nodes

For the geometric regions representing the conduction nodes and cardiac valves, the V-TV and AVN were the most mobile for isolated cardiac motion, with vector centroid displacements >5 mm for 8/10 and 2/10 subjects. For the V-AV, V-PV, V-MV, and SAN, vector displacements were <5 mm for all subjects. Maximum bounding box displacements were >5 mm for the V-AV, V-MV, V-PV, V-TV, and AVN for at least 5/10 volunteers. The SAN was the least mobile, where both centroid vector and bounding box displacements were <5 mm for all subjects.

As a comparison, vector centroid displacement due to isolated cardiac and respiratory motion for the WH was >5 mm for 0/10 and 6/10 subjects, respectively, with maximum bounding box displacement >5 mm for 1/10 subjects and 8/10 subjects.

### 3.3. Statistical considerations

**Figure 4.**
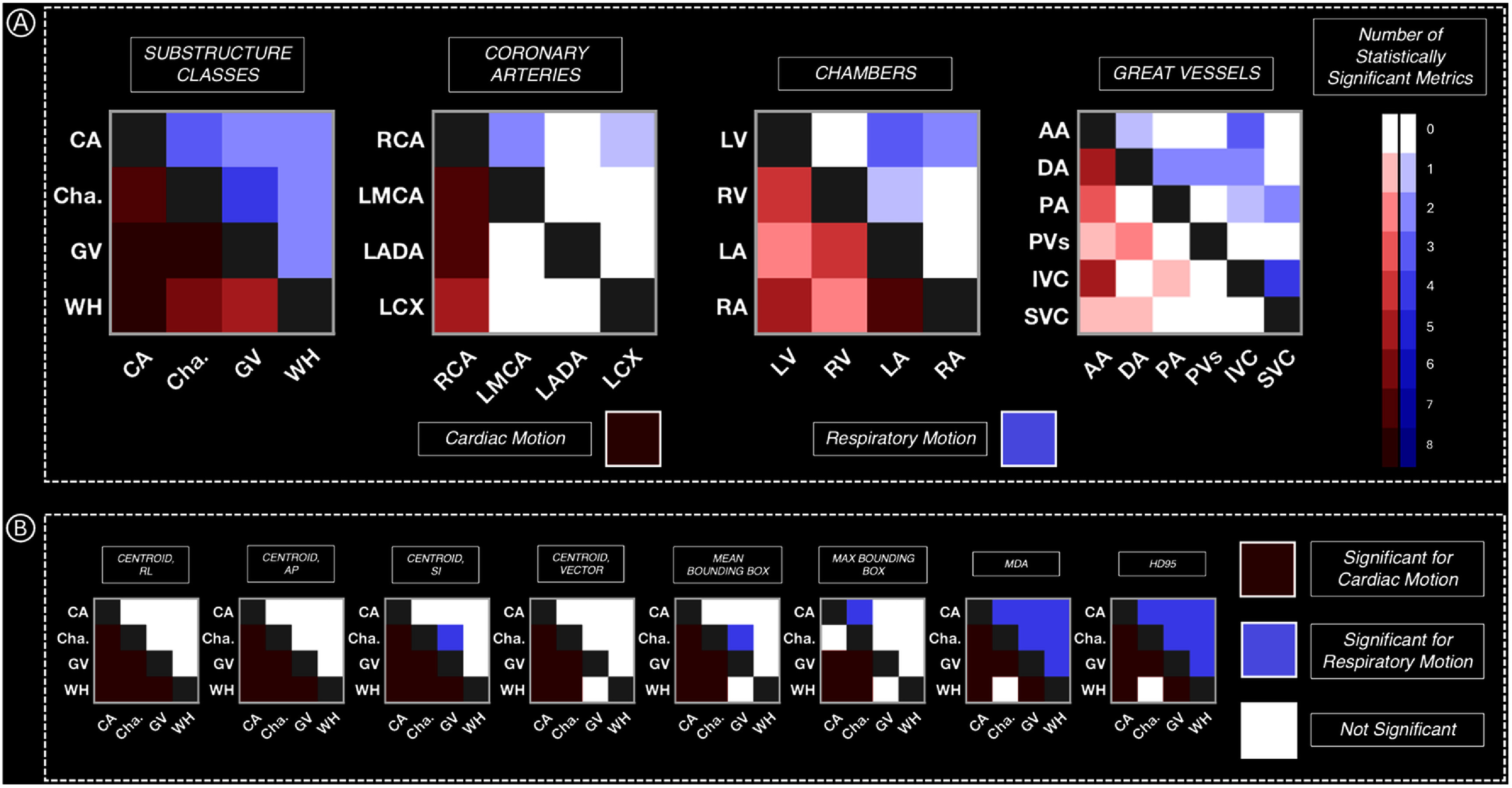
Summary of statistical significance testing results between all pairs of substructure classes, coronary arteries, chambers, and great vessels, for 8 distance metrics (centroid in RL, AP, and SI directions, centroid vector, mean/max bounding box, MDA, and HD95), with results from all 8 tests shown for substructure classes (B). Number of statistically significant metrics for cardiac motion (red) and respiratory motion (blue) is shown. Detailed results of all statistical tests can be found in figure A1. Cha. = Chambers.

A summary of all statistical tests is highlighted in figure 4, with results of specific metrics given in Figure A2. Across substructure groups, significant differences (*P*-value <0.05 for at least 4/8 metrics) in cardiac motion were observed between all pairings of the coronary arteries, chambers, and great vessels, while respiratory motion was significant between the chambers and great vessels. For cardiac motion of the coronary arteries, the RCA was significantly different from the LCX, LMCA, and LADA. All pairings of the LCA were not statistically significant (*P*-value >0.05 for <4/8 metrics) for both cardiac and respiratory motion. For the chambers, statistical differences were observed for cardiac motion between the LA/RV, LA/RA, LV/RV, and LV/RA, while for all pairings of chambers, respiratory motion was not statistically significant For the great vessels, cardiac motion was only significant for the IVC/AA and AA/DA, while respiratory motion was statistically significant between the IVC/SVC Comparing all left substructures (LADA, LMCA, LCX, LV, LA) to all right substructures (RCA, RV, RA), differences in cardiac motion and respiratory motion were significant. Similarly, when comparing substructures near the base of the heart to substructures near the apex, respiratory motion was significant. Comparing the RCA vs right chambers and the LCA vs left chambers, only cardiac motion was statistically significant.

In summary, our novel 5D-MRI strategy was used to conduct comprehensive cardiorespiratory motion assessment in a volunteer cohort. Generally, the RCA demonstrated the largest cardiac excursion, while respiratory motion was greatest for the IVC and LV across our cohort. Following the RCA, cardiac motion was greatest for the LCA, followed by the right chambers, left chambers, and finally the great vessels. Cardiac displacement exceeded 5 mm for the coronary arteries, chambers, V-TV, V-MV, and AVN, and differences in cardiac motion were statistically significant between physiological substructure groupings (i.e. coronary arteries, chambers, great vessels) and the right/left heart. Similarly, substructures near the apex of the heart (i.e. chambers and IVC) demonstrated greater, statistically significant displacements than substructures located near the base (i.e. coronary arteries, AA/DA, PA/PVs, and SVC).

### 3.4. Dosimetric analysis of IRVs

**Figure 5.**
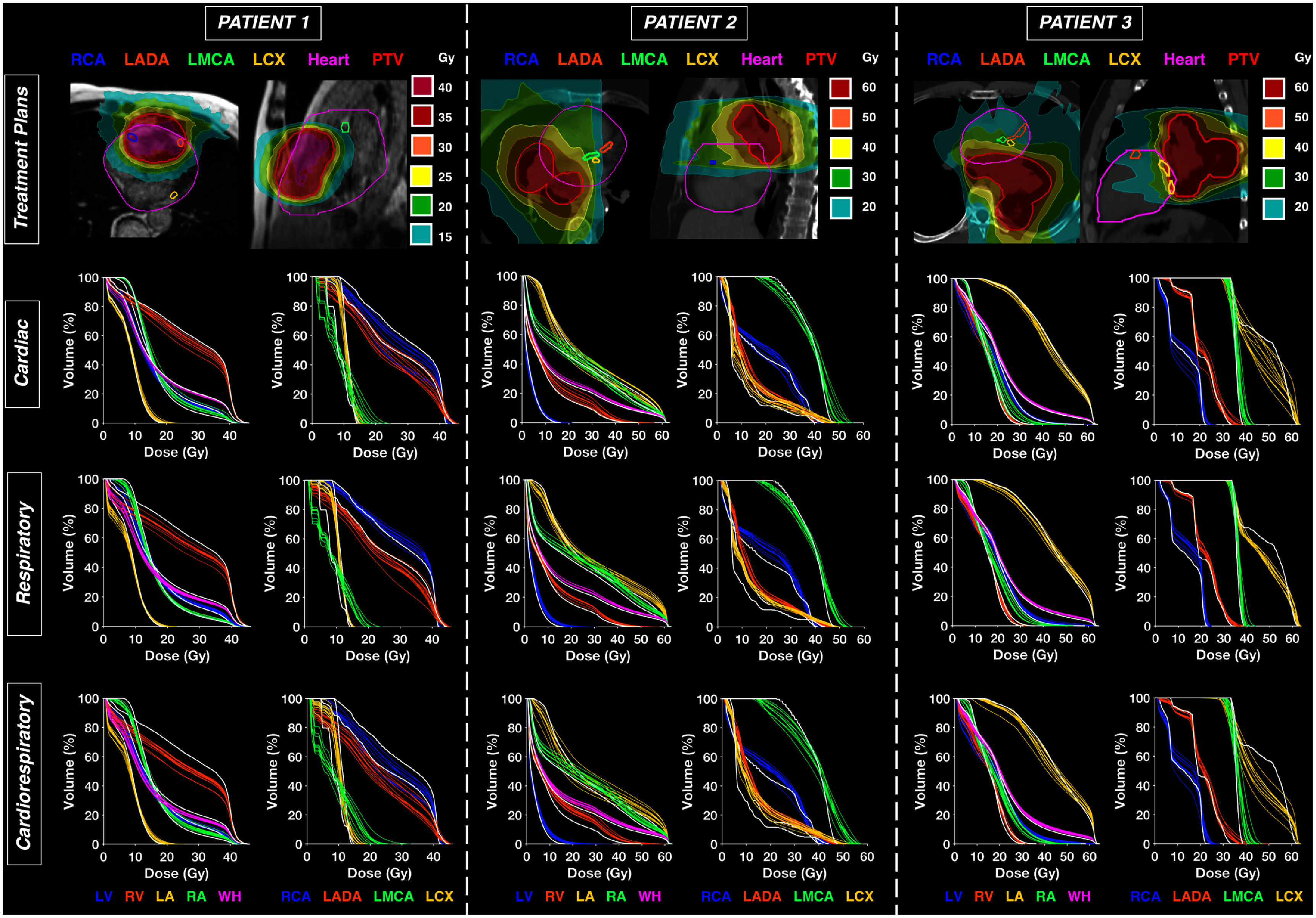
Dosimetric analysis for 5D-MRI-derived internal organ at risk volumes (IRVs) for three patients (top) with (left) a metastasis to the anterior region of the heart, (middle) right-middle lobe lung cancer, and (right) left-lower lobe lung cancer. Dose volume histograms (DVHs, bottom) summarize doses for the coronary arteries and chambers for each patient treatment plan. DVHs for cardiac substructures without added IRVs are shown with a white line, and each individual colored line corresponds to a 5D-MRI-derived IRV from our cohort of 10 volunteers. Isolated cardiac (top), isolated respiratory (middle), and combined cardiorespiratory (bottom) IRVs are shown.

Dose distributions and DVHs are shown for three example thoracic cancer cases in figure 5, where DVHs for reference contours (i.e. cardiac substructures without added IRVs) are highlighted with a white line. Average values and ranges for the change of D_0.03cc_ across all 10 volunteer-derived IRVs for cardiac, respiratory, and combined cardiorespiratory motion with respect to the three stationary plans (i.e. no added IRV, figure 5) are provided in the Supplementary Data (table A4).

Overall, the dosimetric effect of cardiorespiratory motion was dependent on the interplay between tumor location, motion magnitude, and high dose gradient regions. For Patient 1 (figure 5, left), who was treated for a secondary metastasis to the anterior region of the heart, cardiorespiratory motion resulted in decreased dose for the RCA, RV, RA, and LADA compared to the reference contours with no added IRV. These substructures all had significant overlap within the PTV, thus the IRVs encompassing cardiorespiratory motion included lower-dose regions outside the target, resulting in a lower overall dose as observed on DVHs. For the LMCA, which is outside the target yet in a high dose gradient region, increased D_0.03cc_, by 4.4, 5.3, and 10.4 Gy on average for cardiac, respiratory, and cardiorespiratory volunteer-derived IRVs, respectively.

For Patient 2 (figure 5) with right-middle lobe lung cancer, the left coronary arteries (LADA, LMCA, LCX) and RV were located in regions abutting a high dose gradient, thus with increased volumes from volunteer-derived IRVs, the DVHs reflected higher doses. The LV generally received a low dose of <15 Gy, yet for respiratory motion, which is significant for the LV and dominant in the SI direction, D_0.03cc_ increased by 9.0 Gy on average across volunteer-derived IRVs. Due to the tumor’s location superior to the LV, respiratory IRVs included regions with doses up to 20–30 Gy, increasing D_0.03cc_. The RCA had a smaller increase in D_0.03cc_ (Supplementary Data, table A4) and less spread across individual DVH results compared to the left coronary arteries despite being in close proximity to the tumor, likely due to the RCA being located in a region with a smaller dose gradient compared to the LCA, resulting in less dosimetric variation across IRVs.

Patient 3 (figure 5) was treated for left-lower lobe lung cancer that overlapped significantly with the LCX. Similar to the RCA for Patient 1, LCX IRVs for the cardiac, respiratory, and cardiorespiratory motion increased the volume of lower-dose regions outside of the target which yielded a considerable dose spread at intermediate doses within the DVH. However, due to overlap with the tumor, the LCX did not exhibit large changes in D_0.03cc_ compared to the other coronary arteries. For Patient 3, D_0.03cc_ for the RA increased by 6.1, 13.1, and 19.2 Gy on average across volunteer-derived IRVs for cardiac, respiratory, and cardiorespiratory motion, respectively, which was the greatest for any substructure and patient. As the cardiac motion is dominant in the AP and respiratory motion is dominant in the SI directions, both motion trajectories displaced the RA closer to the tumor that was located superior and posterior to the heart. Similarly, D_0.03cc_ increased by 9.9 Gy on average across volunteer-derived cardiorespiratory IRVs for the LADA, due to AP and SI motion and the location of the LADA in a high dose gradient region as shown in figure 5 (top).

Overall, simulations of volunteer-derived IRVs across all treatment plans resulted in increased substructure D_0.03cc_ of up to 14.0, 17.1, and 21.5 Gy for cardiac, respiratory, and combined cardiorespiratory IRVs, respectively. Dosimetric variations for IRVs were highly substructure- and patient-specific. For the LV, which experienced the greatest respiratory motion magnitude in our volunteer cohort, dosimetric variation was greater for respiratory IRVs compared to cardiac IRVs in all plans (Supplementary Data, table A4). Interestingly, the change in D_0.03cc_ across cardiorespiratory IRVs was generally larger for the left CAs compared to the RCA (Supplementary Data, table A4), despite the RCA having the greatest motion magnitude in our volunteer cohort. For comparison, across all evaluated treatment plans and volunteer-derived IRVs, the impact of cardiac, respiratory, and cardiorespiratory motion on D_0.03cc_ for the WH was generally smaller than for cardiac substructures, ranging from 0.0–0.7 Gy (Supplementary Data, table A4). It is important to point out that while differences in the DVH curves from individual volunteer-derived IRVs for the cardiac substructures varied widely, the WH results were not sensitive to motion in the DVH, underscoring the clinical importance of evaluating substructure-specific motion.

### 3.5. Patient motion analysis

**Figure 6.**
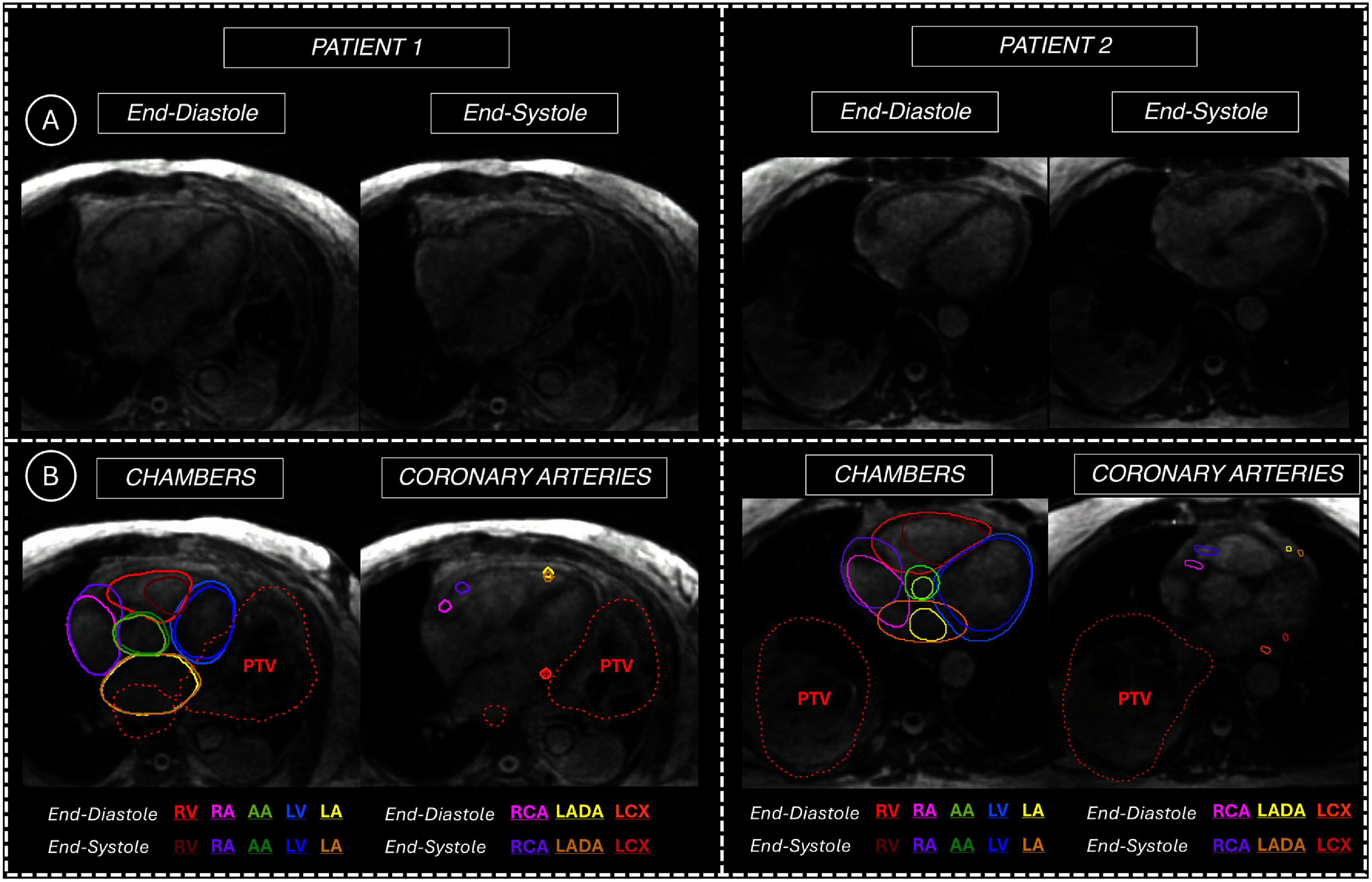
(A) 5D-MRI images acquired from patients with left-sided (left) and right-sided (right) lung cancer at end-inhale for end-diastole and end-systole. (B) Contours for the chambers/ascending aorta and coronary arteries, with the PTV shown with a dashed red line, for both patients.

5D-MRI images and contours for both patients with NSCLC for end-inhale at end-diastole/end-systole are shown in figure 6. For the patient with left-sided NSCLC, vector centroid displacement and maximal bounding box displacements were >5 mm for the right-sided substructures. Given the location and extent of the patient’s disease burden, all other substructures, including the left chambers and LCA (which had centroid or maximal bounding box displacement greater than 5 mm in the volunteer cohort) had displacements less than 3 mm. For the patient with right-sided NSCLC, vector centroid and maximal bounding box displacements were >5 mm for the coronary arteries, chambers, and AA. The RCA was the most mobile substructure, with centroid and bounding box displacements exceeding 19 mm. Centroid and bounding box displacements for all cardiac substructures for both patients are shown in the Supplementary Data (table A5).

## 4. Discussion

This work implemented a novel 5D-MRI workflow to quantify cardiac substructure excursion by decoupling cardiorespiratory motion and providing isolated cardiac/respiratory excursions in a cohort of 10 healthy volunteers. 5D-MRI datasets were acquired in ∼5 min with sufficient image quality to contour all 15 substructures, including the coronary arteries. Centroid and bounding box displacements, HD95, and MDA were used to describe the full extent of motion for each substructure.

Overall, isolated cardiac motion was dominant in the left, anterior, and inferior directions. In a study by Shechter *et al*, which used biplane coronary angiography images to quantify coronary artery motion, cardiac motion was similarly found to be dominant in the left, anterior, and inferior directions (Trzasko *et al* 2011). Across the 10 volunteers in our cohort, cardiac motion was greatest for the RCA with centroid displacements >5 mm for all volunteers and on average, maximum bounding box displacements greater than 1 cm. Our results were consistent with Tan *et al*, who found greater RCA displacement >1 cm compared to the LCA due to cardiac motion as measured with ECG-gated CT (2013). Johnson *et al* used biplane coronary angiography to quantify the 3D displacement of coronary arteries and found that the overall RCA displacement was >2 times larger than the LCA (Johnson *et al* 2004). In the work by Ouyang *et al* which used cardiac-gated CT scans from a cohort of 10 patients to measure cardiac motion, centroid displacements were generally <5 mm for cardiac substructures although some patients had RCA, RV, and LADA displacements >5 mm (2020), which showed some consistency with our 5D-MRI findings.

Centroid excursion due to respiratory motion was generally >5 mm for cardiac substructures, with average bounding box excursions exceeding 5 mm for all substructures (table 1). In our volunteer cohort, the LV and IVC were the most mobile structures due to isolated respiratory motion. Other studies have suggested that respiratory motion is greater for the apex compared to the base of the heart (Shechter *et al* 2006, Liang *et al* 2024), which was also observed here. The question of whether a single margin is sufficient for all cardiac substructures has been previously explored by Yang *et al* (2023), where margins ranging from 0.9 to 6.6 mm were needed to fully compensate for cardiac motion of LV segments when planning esophageal and thoracic radiotherapy treatments. Li *et al* (2018) found that displacements due to cardiac motion between the right and left coronary arteries were statistically significant and require individualized margins for radiotherapy planning. The simulated dosimetric analysis presented in figure 5/table A4 also supports this conclusion, where changes in WH D_0.03cc_ for subject-specific IRVs were relatively insensitive (0.0–0.7 Gy) compared to the other cardiac substructures (up to 21.5 Gy). Our statistical analysis revealed significant differences in cardiac and respiratory motion between the WH and all other substructure groups (i.e. coronary arteries, chambers, and great vessels), indicating a substructure-specific approach is needed for robust planning (figure 4). Miller *et al* found that the IVC and RCA experienced the largest respiration-induced displacement, using 4D-CT and T2-weighted MRI to quantify respiratory motion of cardiac substructures (2022). While the RCA was found to have less respiratory motion than the IVC or LV in our cohort, our coronary artery segmentations included only the proximal regions, while Miller *et al* used the full length of the arteries (2022). Since these segments are closer to the base of the heart than the medial/distal regions of the coronary arteries, it follows that respiratory motion would be smaller. Excursion due to respiratory hysteresis was <5 mm for all substructures on average across subjects, for both centroid and bounding box excursions. However, for some substructures in some volunteers, there were instances where bounding box and centroid excursion exceeded 5 mm (table A3), indicating that motion due to respiratory hysteresis is subject specific and may need to be considered in free-breathing treatment schemas.

Regions representing the valves and nodes exhibited varying magnitudes of cardiac and respiratory motion. The AVN and V-TV had average centroid displacements of >5 mm, while maximum bounding box displacements exceeded 5 mm for the AVN, V-TV, and V-MV. These substructures are both highly mobile and correlated with radiation-induced valvular heart disease (Lee and Hahn 2023) and arrhythmias (Atkins *et al* 2024), and may therefore require additional consideration during treatment planning.

Across three thoracic cancer treatment plans (figure 5), dosimetric variation across volunteer-derived IRVs for cardiac substructures was highly dependent on motion magnitude, tumor proximity, and high dose gradient regions. Generally, the substructures with large motion magnitudes, such as the coronary arteries for cardiac motion or LV for respiratory motion, experienced the greatest dosimetric variation. However, the proximity of cardiac substructures to the target greatly influenced the dosimetric impact of the motion. For example, the RCA, which was the most mobile substructure in the volunteer cohort, had smaller changes in D_0.03cc_ compared to the left CAs, and the average variation in D_0.03cc_ for the RA was <1 Gy for Patients 1 and 2 yet up to 19.2 Gy for cardiorespiratory motion in Patient 3 (Supplementary Data, table A4). Chin *et al* explored the dosimetric impact of respiratory motion in lung cancer radiotherapy, delineating the cardiac substructures on 4D-planning-CT to quantify respiratory motion (2024). Between different respiratory phases, variations in D_0.03cc_ of 2.4–22.6 Gy for the coronary arteries were observed (Chin *et al* 2024), which are similar to our results for isolated respiratory motion. Li *et al* (2024) explored the effect of cardiorespiratory motion of the chambers on SBRT plans for the treatment of VT and found greater dosimetric variation for respiratory IRVs compared to cardiac IRVs. Similarly, Omidi *et al* (2023) observed a greater dosimetric impact of respiratory motion on LV dose compared to cardiac motion for lung cancer radiotherapy. In our analysis, we similarly observed greater dosimetric variation for respiratory IRVs of the cardiac chambers, except for the RV for Patient 2, which is a right-sided lung cancer case. Our simulated dosimetric results indicate that the impact of cardiorespiratory motion on cardiac substructure dose is largely dependent on tumor location and substructure motion magnitude, suggesting the necessity of a substructure-specific, patient-specific approach to enable cardiac-spared treatment planning. The WH was insensitive to cardiorespiratory motion compared to individual substructures, further indicating that a substructure-specific approach to motion assessment may be advantageous.

Our simulated dosimetric analysis assumed that each substructure inhabits the expanded IRV throughout treatment delivery, which simplifies the complex dosimetric blurring that occurs because of intra-fraction motion uncertainties. For a subset of cardiac substructures (e.g. WH, great vessels) with relatively small displacements due to cardiac and respiratory motion, the dosimetric impact is likely to be averaged out over many cardiac cycles and fractions. However, for substructures with large displacements (e.g. coronary arteries, right chambers) or substructures in close proximity to the PTV (e.g. LCX or LV for left-sided NSCLC), cardiac and respiratory motion may lead to clinically relevant dosimetric differences throughout treatment delivery across fractions. In hypofractionated treatment schema (e.g. Patient 1, figure 5), the impact of cardiac and respiratory motion may need greater consideration, due to less blurring of dose across a fewer number of fractions. Once our 5D-MRI workflow is expanded to patient cohorts, future work to derive probability density functions (PDFs) from 5D-MRI for treatment plan optimization and evaluation in manner similar to Bortfeld *et al* (2008) could be explored.

A limitation of our retrospective dosimetric analysis is that IRVs derived from healthy volunteers were applied to treatment plans from patients with thoracic cancers, which may be unrepresentative of the true cardiorespiratory motion patterns in patients undergoing cancer therapies as was observed in our prospective 5D-MRI assessment of cancer patients with reduced excursion due to cancer burden. Therefore, patient-specific assessments should be considered to determine the dosimetric impact of cardiorespiratory motion to facilitate highly effective cardiac substructure dosimetric sparing. Further, by assuming that dose delivery is static instead of time-averaged over many cardiac and respiratory cycles, the clinically delivered maximum dose received by each cardiac substructure provides an overestimation in our simulations. Nevertheless, these data are still informative for potential worse-case scenarios. While outside the scope of this study, future work to account for time-averaged dose by deriving a PDF from 5D-MRI phases can be explored.

For the patients with locally advanced NSCLC who were scanned with 5D-MRI, image quality was sufficient for contouring the cardiac substructures, and due to the post-contrast acquisition, increased contrast was observed between the LV myocardium and blood pool compared to the non-contrast volunteer scans (figure 6). In addition to the subject-specific motion amplitudes observed in the volunteer cohort, cardiac substructure motion was also found to be highly variable between the two patients with NSCLC. For motion assessment, right-heart substructures had centroid and bounding box displacements exceeding 5 mm in both patients, which was similarly observed in the volunteer cohort. However, for the left-sided NSCLC case, the LCA and left chambers had centroid and bounding box displacements less than 3 mm, which is smaller than the excursions observed in volunteers and the patient with the right-sided lesion. This is likely due to the location and extent of the gross tumor volume, which overlapped the left heart wall and likely reduced cardiac motion. The observed differences between the two patients further supports that patient-specific margins may be required for cardiorespiratory motion-robust planning, as each patient’s tumor location and extent may further affect cardiac substructure motion. The effect of tumor location on overall magnitude of cardiorespiratory motion can be explored in future work as more patient scans are conducted. Future applications in SBRT and VT are possible, however cardiac doses may be low in the former population and image quality optimization with implanted cardiac devices would be necessary for the latter population.

The results of this preliminary work suggest that both the cardiac and respiratory motion of cardiac substructures differs both with physiological grouping, as well as location within the heart (right vs left heart, heart base vs apex). Previously, cardiorespiratory motion quantification has been evaluated using multi-modality approaches, combining 4D-CT for respiratory and cine MRI sequences for cardiac motion (Omidi *et al* 2023), or combining multiple cine MRI at different respiratory states to quantify cardiorespiratory motion (Liang *et al* 2024). However, multi-modality or multi-acquisition approaches result in longer overall scan time to account for multiple acquisitions that require co-registration that may introduce more uncertainty. Our 5D-MRI workflow uses a ∼5 min acquisition, where all cardiac and respiratory phases are natively registered to each other, which eliminates potential registration error and reduces scan time. Further, as data are derived from a single acquisition, our technique offers the benefits of no temporal mismatch or co-registration uncertainties.

A limitation of our work includes the small sample size (10 healthy subjects, 2 patients with lung cancer) and general skew toward younger, male healthy volunteers. Nevertheless, the cohort also included four subjects over the age of 60, with one having a coronary stent in their LADA and two having locally advanced NSCLC, suggesting applicability of our 5D-MRI workflow in more complex scenarios including cancer cohorts. Further validation will be necessary to confirm that the observed displacements and statistical relationships apply to cancer patient populations. One advantage of our 5D-MRI acquisition is that it is acquired over free-breathing conditions in 5 min, which would be well-tolerated by cancer patient cohorts without added breath-hold requirements for populations who may be unable to comfortably perform DIBH. Another limitation is that our 5D-MRI iterative reconstruction process is performed entirely offline in a research computing environment, taking ∼6 h to reconstruct all 40 cardiac and respiratory phases. For our 5D-MRI workflow to be implemented clinically, reconstruction time could be reduced by reconstructing fewer phases or leveraging GPU acceleration. Another limitation of this feasibility study was that contours were manually delineated, increasing our analysis time. Now that these baseline data are delineated, future work can include training deep learning segmentation algorithms on the 5D-MRI datasets (Kim *et al* 2022, Summerfield *et al* 2024) to further reduce uncertainties and streamline delineation time.

An additional limitation of this work is that the pulse oximeter-based gating was used in the healthy volunteer cohort, due to volunteer comfort and ease of setup (e.g. no skin preparation for electrode placement). Gating via pulse oximeter instead of ECG may introduce greater uncertainty due to delay in the end-systolic triggering window (Bert *et al* 2020). However, since data are acquired throughout the cardiac cycle retrospectively binning the data, the precise timing of the end-systolic window is not as important as with prospective cardiac gating. No meaningful differences in 5D-MRI image quality were observed between gating with ECG and pulse oximeter (Supplementary Data, figure A1), where a volunteer from the cohort was rescanned approximately 1 year after the initial scan using ECG-based gating. However, for the two patient scans, ECG-based gating was implemented following the clinical protocol with similar results. These results illustrate that our 5D-MRI workflow can use either pulse oximeter or ECG signals (if electrodes are implemented) for cardiac sorting, allowing for flexibility based on the clinical endpoints and the technology available. Another limitation of our work is the use of bellows as a surrogate for respiratory motion during reconstruction, which may introduce additional error as its use as an external surrogate for internal respiratory motion may not be accurate. However, several studies have shown that bellows signal is strongly correlated to diaphragm motion (Santelli *et al* 2011, Lauria et al., 2021, Madore *et al* 2006). Our respiratory binning is phase-based instead of amplitude-based and uses a moving average strategy to calculate bin thresholds, so the motion amplitude reading of bellows is less critical to the end result. Further, using bellows-based respiratory gating has been shown to yield faster MRI acquisition times (Santelli *et al* 2011). The implementation of navigator-based gating is non-trivial due to the necessary addition of a free induction decay acquisition within the pulse sequence (Maatman *et al* 2024), but its inclusion could be a potential direction for future work.

Potential sources of error associated with contouring include delineation error, inter-observer variability, and potential blurring due to data binning during reconstruction. However, these sources of error were minimized by acquiring other clinical scans as a reference (i.e. 3D-Nav) to augment segmentation accuracy, verification of contour accuracy by a cardiac anatomy expert, and using a small respiratory bin width of 10%. To balance the tradeoff between scan time (∼5 min) with the number of radial acquisitions at the 1.56 mm isotropic image resolution needed to resolve cardiac substructures, the resultant 5D-MRI datasets will have low SNR compared to diagnostic-quality cardiac MRI typically acquired at a thicker slice thickness. Nevertheless, our 5D images are of sufficient quality to contour larger structures (i.e. cardiac chambers, great vessels). The reconstructed resolution of our 5D-MRI images (1.56 mm isotropic) may limit the accurate delineation of small vessels (i.e. the coronary arteries). However, the image resolution of our 5D-MRI is comparable to recent state-of-the-art MR angiography sequences, where a 1.2–1.5 mm isotropic image resolution has been noted to be sufficient for coronary artery visualization and delineation (Fok *et al* 2021, Craft *et al* 2025). To aid with the delineation of coronary arteries, a supplemental scan (3D-Nav) was acquired, however, in the majority of cases, the coronary arteries were able to be delineated directed on 5D-MRI without added reference of the 3D-Nav. The inclusion of fat suppression (Koktzoglou and Edelman 2018) would improve signal from the blood pool and potentially allow for improved delineation of cardiac substructures, however the implementation with radial sampling is outside the scope of this work and can be a topic of future study. Data shown in patients (figure 6) highlight how acquiring 5D-MRI post-contrast enhanced the contrast of the blood pool and coronary arteries, allowing for improved delineation of cardiac substructures.

In our 5D-MRI workflow, reconstructions were performed for 4 respiratory phases, which is fewer than the typical 10 bins used for 4D-CT (Abdelnour *et al* 2007). However, studies have demonstrated statistical equivalence between using 4D-CT with fewer respiratory phases (i.e. 4) and 4D-CT with 10 phases for motion characterization (Cao *et al* 2012, Yeo and Kim 2013, Koksal *et al* 2021). While reconstruction for 10 respiratory phases is possible in our workflow, our centroid and bounding box data suggest that cardiac substructure motion due to respiratory hysteresis is generally insignificant (<5 mm), and the addition of more respiratory phases would further degrade image quality due to increased data starvation. Therefore, 4 respiratory phases were chosen for reconstructions to capture the extreme respiratory and diastolic phases. Additionally, the use of 4 respiratory phases has been implemented for respiratory motion-resolved free-breathing cardiac MRI, where the balance between data starvation and scan time is of more concern (Hu *et al* 2013, Piccini *et al* 2017).

This preliminary work was concerned with quantifying isolated cardiac and respiratory motion from a single acquisition. Our 5D-MRI strategy is akin to a single 4D-CT acquisition used for treatment planning, where the underlying purpose of the sequence is to derive IMs, used for deriving either IRVs (for OARs) or ITV (for PTV, in the case of 4D-CT). Comprehensive PRV derivation would require multiple time points and a larger volunteer cohort to calculate setup uncertainties and determine population averages, which may be possible in future work as patient data becomes available but is outside the scope of this work. Inter-subject variability in substructure cardiac and respiratory-induced motion was observed in this study, suggesting that individualized margins may be necessary to enable the most robust cardiac sparing. Further investigation applying our novel 5D-MRI workflow to cancer patient cohorts can be used to determine population-based PRVs for individual substructures and substructure groupings. Future work includes extension of the 5D-MRI workflow for comprehensive motion quantification in an upcoming cardiac-sparing trial for thoracic cancer patients and possible application to patients undergoing radioablation of VT.

## 5. Conclusion

Our 5D-MRI workflow has been successfully demonstrated to decouple and quantify cardiorespiratory motion for coronary arteries, chambers, and great vessels via a 5 min free-breathing acquisition. Across our volunteer cohort, cardiac motion was highly substructure specific. The RCA, LCA, and chambers exhibited significant cardiac motion (>5 mm). Respiratory motion was dominant in the superior–inferior direction and was >5 mm for all substructures, with substructures near the apex of the heart experiencing greater respiratory motion than the base. Statistically significant differences were observed between the right/left heart and coronary arteries/chambers/great vessels. Differences between the apex/base of the heart were statistically significant. Variation in radiation dose to 5D-MRI-derived IRVs accommodating cardiorespiratory motion was greatly dependent on tumor location and substructure motion magnitude in three simulated example cases. 5D-MRI was demonstrated for two patients with NSCLC, where left-heart substructure motion varied between the patients due to tumor location. Future work includes expanding our 5D-MRI workflow to patient cohorts for comprehensive margin assessment to support cardiac sparing in thoracic cancer treatments with future applications to VT SBRT.

## Data Availability

The data cannot be made publicly available upon publication because the cost of preparing, depositing and hosting the data would be prohibitive within the terms of this research project. The data that support the findings of this study are available upon reasonable request from the authors. Supplementary Data 1 available at https://doi.org/10.1088/1361-6560/ae5752/data1.
